# Systems Biology and P4 Medicine: Past, Present, and Future

**DOI:** 10.5041/RMMJ.10112

**Published:** 2013-04-30

**Authors:** Leroy Hood

**Affiliations:** President, Institute for Systems Biology, Seattle, WA, USA

**Keywords:** P4 medicine, systems medicine, systems biology, personalized medicine, disease stratification, patient stratification, systems-driven diagnostics

## Abstract

Studying complex biological systems in a holistic rather than a “one gene or one protein” at a time approach requires the concerted effort of scientists from a wide variety of disciplines. The Institute for Systems Biology (ISB) has seamlessly integrated these disparate fields to create a cross-disciplinary platform and culture in which “biology drives technology drives computation.” To achieve this platform/culture, it has been necessary for cross-disciplinary ISB scientists to learn one another’s languages and work together effectively in teams. The focus of this “systems” approach on disease has led to a discipline denoted systems medicine. The advent of technological breakthroughs in the fields of genomics, proteomics, and, indeed, the other “omics” is catalyzing striking advances in systems medicine that have and are transforming diagnostic and therapeutic strategies. Systems medicine has united genomics and genetics through family genomics to more readily identify disease genes. It has made blood a window into health and disease. It is leading to the stratification of diseases (division into discrete subtypes) for proper impedance match against drugs and the stratification of patients into subgroups that respond to environmental challenges in a similar manner (e.g. response to drugs, response to toxins, etc.). The convergence of patient-activated social networks, big data and their analytics, and systems medicine has led to a P4 medicine that is predictive, preventive, personalized, and participatory. Medicine will focus on each individual. It will become proactive in nature. It will increasingly focus on wellness rather than disease. For example, in 10 years each patient will be surrounded by a virtual cloud of billions of data points, and we will have the tools to reduce this enormous data dimensionality into simple hypotheses about how to optimize wellness and avoid disease for each individual. P4 medicine will be able to detect and treat perturbations in healthy individuals long before disease symptoms appear, thus optimizing the wellness of individuals and avoiding disease. P4 medicine will 1) improve health care, 2) reduce the cost of health care, and 3) stimulate innovation and new company creation. Health care is not the only subject that can benefit from such integrative, cross-disciplinary, and systems-driven platforms and cultures. Many other challenges plaguing our planet, such as energy, environment, nutrition, and agriculture can be transformed by using such an integrated and systems-driven approach.

## INTRODUCTION

An old Indian story talks about a group of blind men coming across an elephant. Each of the blind men touched a different part of the elephant and gave a description of what he believed an elephant was. The first person touched the elephant’s trunk and claimed the elephant to be a snake. The second person touched the elephant’s leg and declared the elephant to be a tree trunk. Then the last person came forward, touched the elephant’s ear and positively identified the elephant to be a sail. Based on the blind men’s confined level of interaction with the elephant, their observations made sense. However, if they had collaborated and holistically studied the elephant, its true structure would have become apparent. Understanding complex systems such as the human body can also benefit from the same type of closely interactive collaboration. For many years, biologists have been studying specific proteins and molecular pathways individually, describing local interactions and perturbations in detail. Indeed, understanding the individual components is an important first step, but, to truly understand complex biological systems, an integrated approach must be taken.^1^

The high-throughput biological instrumentation of today, so crucial for personalized medicine, was invented due to a paradigm change in conceptualizing biological research. The hybridization of engineering and biology and the fertile cross-talk between engineers and biologists in the Hood laboratory in the period from 1970 to 1995 produced five different instruments for synthesizing, detecting, and sequencing DNA as well as synthesizing and sequencing proteins.^2^–^4^ Several of these inventions, especially the automated DNA sequencer and the automated DNA synthesizer, made the sequencing of the complete human genome possible and transformed how molecular biology was executed. The genome project was hotly debated at the time.^5^ On the one hand, it was technically feasible, but on the other hand, it was incredibly expensive and arguably an example of the wasteful “big science.” Moreover, due to its very repetitive nature the critics argued that no scientist of stature would participate. In addition, with the genome being full of “junk” sequences, why sequence the genome at all? Eventually, the human genome project did take off and was even completed ahead of schedule and below budget due to the successful integration of different disciplines. Each of the critics’ arguments turned out to be fundamentally flawed.^3^–^6^

## THE ESTABLISHMENT OF THE INSTITUTE OF SYSTEMS BIOLOGY

**Figure 1.**
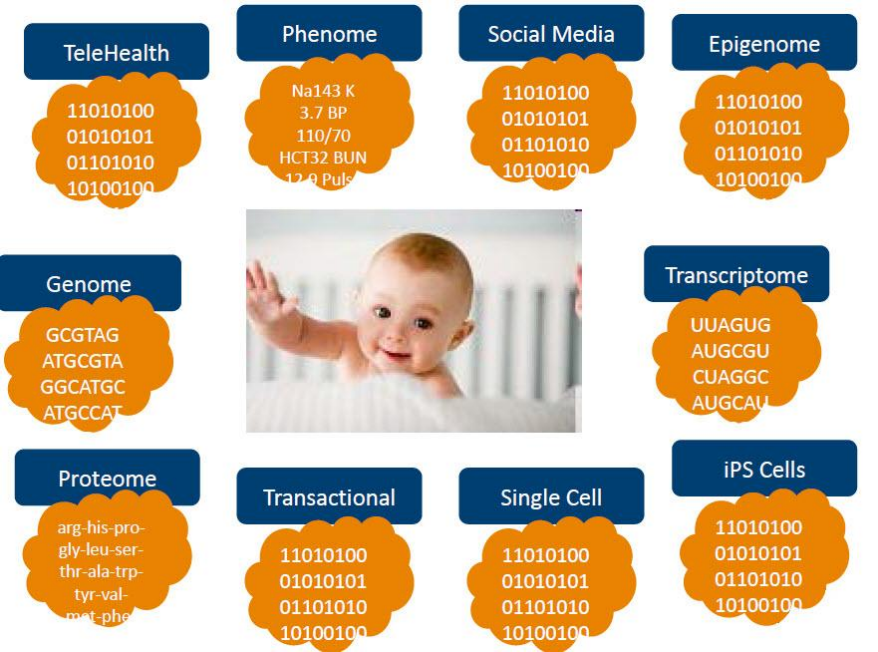
**In 10 years each individual will be surrounded by a virtual cloud of billions of data points—P4 medicine.** Representative examples of the billions of data points that will surround each individual in the near future. From Hood and Flores, New Biotechnology 2012;29(6):613–24, with permission.

Given the rapid advances in technology and systems-driven strategies for personalized health (see below), each one of us will be surrounded by a virtual cloud of billions of data points within a short period of time (Figure 1). Our genome, as well as multiple proteomes, multiple transcriptomes, multiple gut metabolomes, and other personalized data sets obtained at different points in our lives, will be readily available at affordable prices for each individual. The major problem and daunting challenge for medicine will be to find the significant signals within this enormous amount of individual data and enhance the signal-to-noise ratio. In addition, the highly heterogeneous data will have to be integrated into predictive models which will focus on the well-being of the individual. This is not a trivial task by any measure. In order to succeed in understanding a highly complex organism such as the human body, a systems-driven, cross-disciplinary environment will be a fundamental necessity for the biology of the future.^3^,^7^,^8^

The Department of Molecular Biotechnology (MBT) at the University of Washington Medical School was such a cross-disciplinary department from 1992 to 2000. Within a short period of 8 years, the researchers at this department pioneered fundamental new techniques in the emerging field of proteomics, created the software that fueled the genome project, developed a revolutionary multi-parameter high-speed cell sorter, and pioneered the ink-jet DNA synthesizer that could both synthesize thousands of DNA fragments and generate DNA arrays with hundreds of thousands of DNA fragments.^3^,^4^ We wished to build an Institute for Systems Biology in addition to the cross-disciplinary platform of MBT. However, bureaucracies at large institutions, honed by the past, are often barely capable of dealing with the present, let alone the future. Frustrations with different university bureaucracies were the impetus for creating the independent, non-profit Institute for Systems Biology (ISB) in Seattle. The ISB was established as a non-traditional institution, where scientific collaboration could take place across disciplines and where biologists and other scientists, along with technologists, could freely commingle, creating a milieu in which the cross-pollination of ideas was the rule and not the exception.^3^ It has taken us more than 10 years to create the cross-disciplinary culture where scientists speak one another’s languages and they can work together effectively in teams.^8^ Our cross-disciplinary culture is very much driven by the idea that leading-edge biology necessitates the need to invent new technologies (and thus open new areas of data space for exploration) and that these new technologies mandate the development of new mathematical and computational analytical tools (e.g. the ISB mantra, the “holy trinity,” is “biology drives technology drives computation”). This cross-disciplinary, systems-driven platform and culture also foster innovation because the “holy trinity” creates new technologies, new analytical tools, and finally new concepts—and these have fueled significant company creation by ISB.^2^,^3^

## SYSTEMS MEDICINE

**Figure 2.**
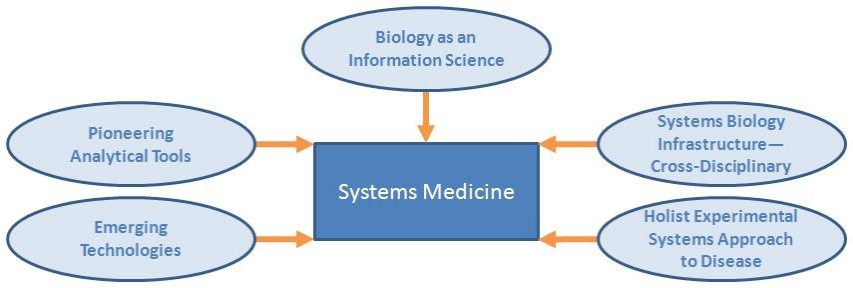
**The elements that will allow systems medicine to tackle deciphering biological complexity.** These represent the strategies, technologies, and analytical tools that will enable the implementation of systems biology and P4 medicine.

Establishing the ISB was only the first step in creating a systems medicine approach to health care. Other concepts had to be integrated in order to utilize complex biological systems for predictive, preventive, personalized, and participatory (P4) medicine.^1^–^3^,^7^ These concepts included: treating biology as an information science, creating a cross-disciplinary, systems biology infrastructure and culture, designing an experimental holistic integrative approach to biology along with developing new technologies (and improving the old technologies) that will allow the exploration of new dimensions of patient data space, and, lastly, inventing analytic tools that will analyze and interpret all the information generated by the newly developed technologies (Figure 2).

**Figure 3.**
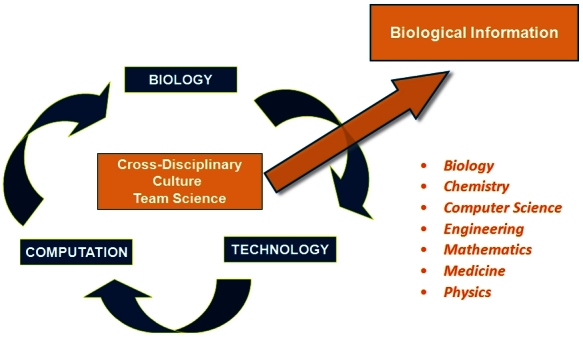
**Holy trinity of the biological cross-disciplinary culture—“biology drives technology drives analytical tools revolutionizes biology.”** Biological breakthroughs require technological innovations which in turn necessitate new computing tools. New technological and computing tools, in turn, allow for the exploration of new biological frontiers. Adapted from Hood and Flores, New Biotechnology 2012;29(6):613–24, with permission.

The nature of the infrastructure needed for creating systems biology and systems medicine is one that encompasses widely disparate cross-disciplinary backgrounds.^3^,^8^ The human infrastructure includes biologists, chemists, computer scientists, engineers, physicists, and mathematicians. Cross-disciplinary environments cannot be divided into separate departments as is done in most universities. They must be in close proximity to each other, where random collisions create new opportunities and new ideas. This is how the Institute for Systems Biology is structured, where productive cross-disciplinary cross-talk is the norm and not the exception. Leading-edge biology dictates and mandates the creation of new technologies. These technologies in turn specify the nature of the new analytic tools that must be created to handle the information (Figure 3). As noted earlier, the situation in which biology drives technology which in turn drives computation can only work in the context of a cross-disciplinary environment where scientists learn to speak each other’s languages and learn to work effectively together in teams.

### Biology as an Information Science

Biology can be defined as an informational science. This definition is important since it gives us a conceptual framework to deal with biological complexities. There are two types of information in biological systems: the digital information of the genome and the environmental information which consists of signals brought from outside the genome. These data are integrated in the organism to create either the normal or the diseased phenotype. Two information-handling systems connect the two types of biological information with the phenotype. The first system is made up of biological networks that capture, transmit, modulate, and finally pass the information off to the second system. The second system consists of both simple and complex molecular machines which execute the commands given by the signals they receive. The temporal and spatial dynamics of the two systems is of crucial importance for the understanding the homeostasis of the organism.

Biological organisms consist of interconnected biological networks of networks, both within and between cells. To truly understand complex biological phenomena, they must be studied in the context of this network complexity. A holistic, integrative or systems approach to biology and medicine can be explained by a simple analogy. In order to understand how a radio converts electromagnetic waves into sound waves, the first step would be to compile a list of the components. Then the components would be studied individually to ascertain what each component does independently. After understanding the individual parts, the next step would be to assemble the parts into circuits and then understand individually and collectively how the circuits convert radio waves to sound waves. Similarly, for the last 40 years, biologists have focused on individual genes and proteins. The genome project supplied the entire parts list of genes and, by inference, proteins. Similar to the radio, organisms have circuits and biological networks, and these networks handle information and process it. The dynamics of these processes is crucial for understanding the body’s normal healthy state, as well as the initiation and progression of the disease.

**Figure 4.**
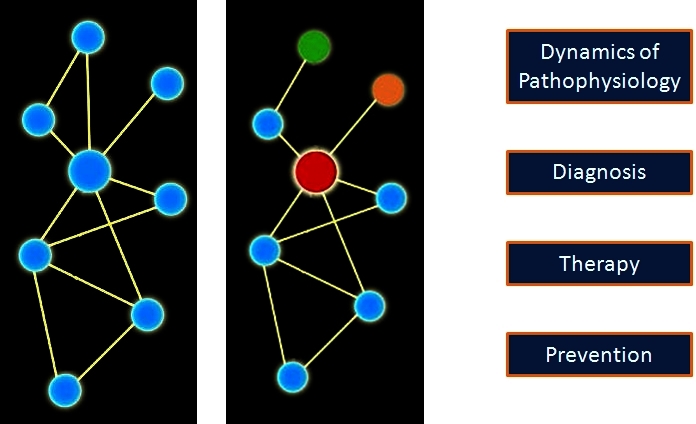
**A schematic view of a normal (left) and disease-perturbed network (right).** Both nodal points (colored balls) and edges (lines attaching the balls) change in disease, as indicated by changing colors indicative of changing levels and the disappearance of an edge. The nodes and edges change dynamically with disease progression. Understanding the dynamics of networks permits one to understand the dynamics of the pathophysiology of the disease and think about new approaches to diagnosis, therapy, and even prevention. Taken from http://prion.systemsbiology.net/page/PosterView/display/poster_id/14

**Figure 5.**
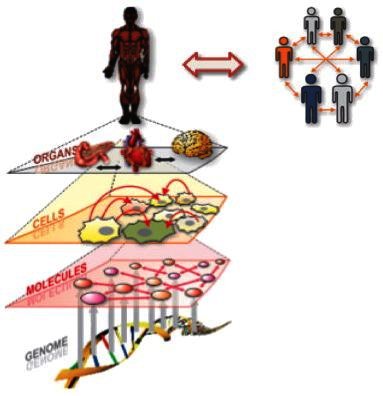
**Systems medicine—the network of networks**. A schematic view of the different levels of networks and their interconnectivity in the body. Networks organize, integrate, and model data to increase enormously the signal-to-noise ratio. From Hood and Flores, New Biotechnology 2012;29(6):613–24, with permission.

In a simplified model of a systems view of disease, one or more biological circuits becomes disease-perturbed, either genetically and/or environmentally, thus altering the envelope of information expressed by that disease-perturbed circuit (Figure 4). The altered envelope of information explains the pathophysiology of the disease and provides new insights into diagnosis, therapy, and prevention of the disease. In reality, there is not only one intrinsic network but networks of intrinsic networks: genetic networks, molecular networks, cellular networks, organ networks, and, finally, the assembly of the networks which operate in the context of the individual. In addition, there are extrinsic social networks that modify our environment. Both intrinsic and extrinsic networks must be taken into account to get the true systems view of disease (Figure 5).^7^

Integrating all the networks and understanding how they collectively respond to the digital and environmental signals is a daunting task. One way to simplify this task is to suppose that these networks are fractal in nature. Therefore, all the hierarchical levels of organization are similar in structure. If this assumption is valid, we can study networks at an accessible level and make inferences about how they operate at the higher and less accessible levels.

### Holistic Experimental Systems Approach to Disease

**Figure 6.**
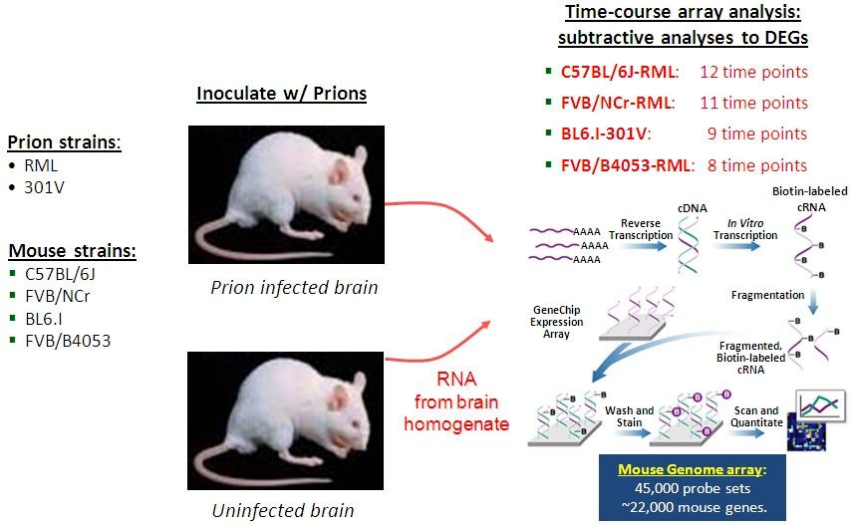
**A schematic view of the mouse prion experiment.** Two different prion strains and four different mouse inbred strains are used. For both control and experimental animals transcriptomes are determined across the progression of the disease.

An example of a systems view of disease is the prion-induced neurodegenerative disease. Using a mouse model (Figure 6),^9^,^10^ we initiated the disease by injecting infectious prion particles into the brain. Normally, it takes 22 weeks for the disease to run its course in mice. During the 22-week period, we analyzed the complete transcriptome of the brain at 10 different time points. At each time point, we subtracted the transcriptomes of the normal mice from the transcriptomes of the diseased mice, thus ending with only the genes that were differentially expressed (DEGs). However, even after subtracting the normal genes from the diseased mice, we were left with about a third of the mouse brain genes that were differentially expressed. Normally, about 17,000 genes are active in a mouse’s brain, and in this case about 7,400 were differentially expressed—thus representing an enormous signal-to-noise challenge. Noise can be divided into two types: technical noise that comes from generating and manipulation of data, and biological noise that arises as a consequence of the different biologies operating in an organ such as the brain. If you assay a phenotype such as the brain transcriptome, the result is almost always the sum of a number of different biologies. If only one specific phenomenon is of interest, such as neural degeneration, all the other biological phenomena must be subtracted away.

Different strains of mice were created to subtract away the non-neurodegenerative phenomena from the roughly 7,400 genes that were differentially expressed in the prion-diseased mouse brain. For instance, a mouse which was a double knock-out for the prion gene was created, so, when injected with infectious prion particles, it did not contract the disease. However, its brain transcriptome changed, reflecting DEGs arising from other biologies that could be subtracted away. This subtraction process was repeated with the other carefully selected mouse strains that reflected other irrelevant biologies that could be subtracted away as well. After eliminating all the non-neurodegenerative phenomena, the slightly more than 300 genes that were left encoded the core of the neurodegenerative response.

**Figure 7.**
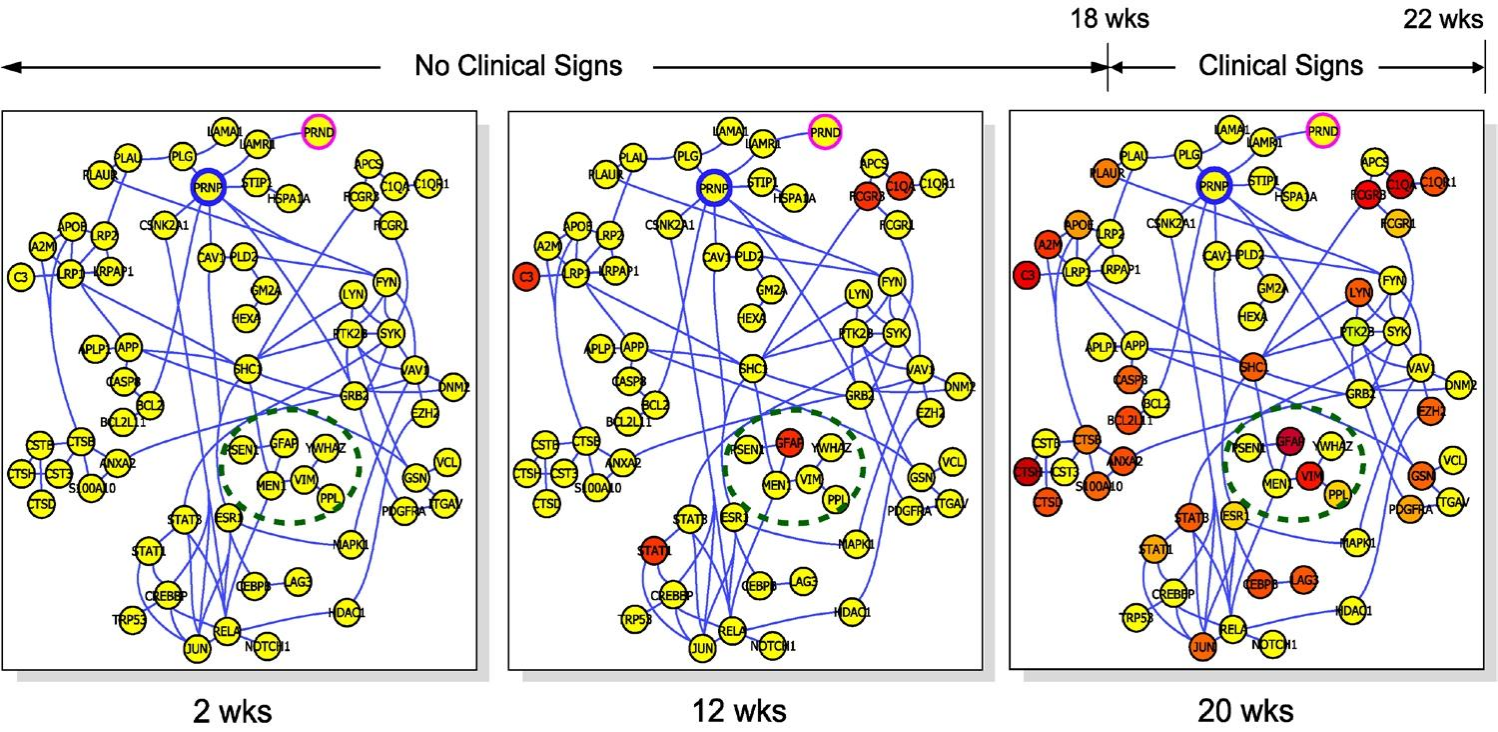
**A schematic view of networks becoming disease-perturbed as the prion disease advances.** The prion disease progression takes about 22 weeks. The red balls indicate transcripts that have been elevated in the prion-infected brains. From Hood and Flores, New Biotechnology 2012;29(6):613–24, with permission.

**Figure 8.**
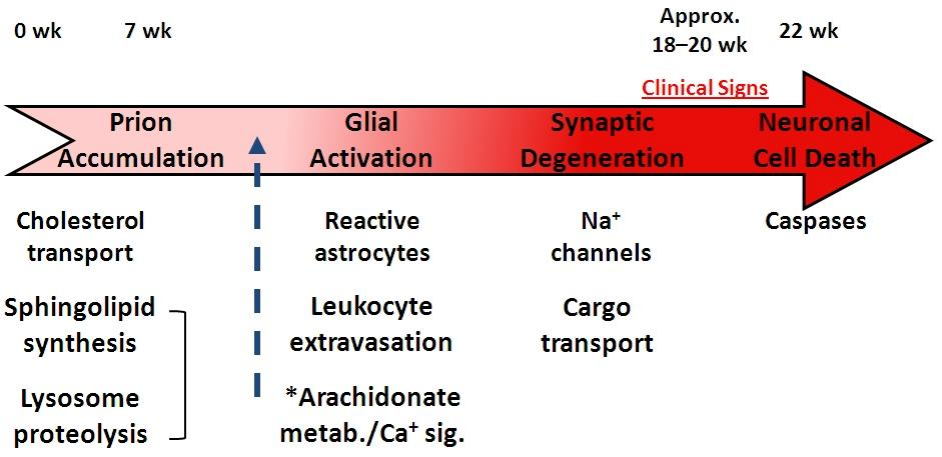
**An example of the biological networks that become successively disease-perturbed as prion disease progresses.**

Four basic processes delineate the dynamic histopathology of this disease: Prion accumulation and replication, glial activation, and two different forms of neurodegeneration: synaptic degeneration and neuronal cell death. The identified genes were mapped across multiple time points and across the identified interaction networks that encode for these four processes. The picture that emerged was that in the beginning of the disease both normal and diseased mouse networks were the same (Figure 7). However, as the disease progressed, more and more networks were recruited into the disease state. One other very striking observation was the temporal sequential perturbation of the four major identified networks to the diseased state.^9^–^10^ The disease started in the most unique network of prion accumulation and replication and then progressed to the other networks (Figure 8). Therefore, the strategy for attacking such a disease from the point of view of diagnostics and therapeutics would be from the initially affected network.

Several insights were gained by using this systems-based model. First, two-thirds of the 300+ genes mapped into the four prion disease networks. Second, the remaining 100 genes identified six new smaller networks that had not been previously associated with the disease process. Third, not only were the four main networks sequentially perturbed in the disease, but all 10 networks became sequentially disease-perturbed. Finally, the dynamics of the 10 networks could explain virtually every aspect of the pathophysiology of the disease, giving fundamental new insights into both potential for therapy and diagnosis of the disease.

### Proactive Diagnosis

Diagnosis is an area that can highly benefit from the systems-based approach. If proteins from a diseased organ or blood are compared to the normal state, many differences will be found. However, the overwhelming majority of these differences represent noise, and without a systems approach it is extremely difficult to sort out the signal from the surrounding noise. To reduce the noise, two working assumptions are used: first, that blood bathes all organs, both the accessible and the inaccessible ones; second, that all organs secrete proteins into the blood. A fraction of the proteins that are secreted into the blood from each organ are uniquely synthesized in that organ and are therefore denoted “organ-specific proteins.” These proteins with their unique molecular addresses can be used to determine the location of a disease.

In order to create organ-specific fingerprints, we generated assays using targeted mass spectrometry for roughly 100 proteins in both mouse and human for two different organs, the liver and the brain.^11^–^14^ For each healthy individual, every one of the 100 or so brain-specific proteins found in the blood has a specific set of concentrations. If a neural disease is initiated, proteins from the networks that have become perturbed by the disease will alter their concentrations in the blood. They will alter their concentrations in the blood in a way that uniquely defines each disease because each disease perturbs different combinations of biological networks. Hence, we can distinguish health from disease and also determine which disease by measuring the organ-specific proteins in the blood sample.

To show that this model works, we took 15 murine brain-specific proteins which evenly mapped to four major networks. We then demonstrated from the blood that we can do two things: 1) diagnose the disease eight weeks before any clinical signs were apparent, i.e. early diagnosis; and 2) follow the sequential disease perturbation of the four major networks, i.e. disease progression. This shows that this method can both diagnose and follow the disease by using blood samples only (H. Yoo, personal communication). In addition, this method provides the capability to distinguish between several different neurodegenerative diseases, that is, to stratify these diseases and follow the body’s response to therapy and reoccurrences.

Macromolecules have to meet certain criteria to be considered ideal biomarkers.^12^ First, these markers must exist and be detectable in the blood since the blood is in contact with and bathes all the body’s organs. The blood, which is readily available, gives us access to all the body’s organs, including organs that are ordinarily difficult to access, such as the brain. Second, the group of organ-specific markers must be multi-parametric so that multiple networks and features from each organ can be assessed. Third, the array of biomarkers for each organ will be able to assess all diseases in a given organ simultaneously because they sample many different biological networks. These biomarkers do not necessarily have to be only proteins. Biomarkers can be other biomolecules such as mRNAs, miRNAs, metabolites, or other macromolecules.^12^,^15^ The assays using these markers must always be done in a longitudinal manner, making each person his/her own control for the changing levels of biomarkers in the blood. The longitudinal method of testing will enable one to follow transitions from health into disease.

In addition to organ-specific markers, organelle-specific markers should be sought to reflect direct or indirect network disease perturbations such as cell death. Both cytoplasmic and nuclear proteins can be used for this purpose. Additionally, membrane-cleaved proteins as well as secreted proteins will provide fundamental insights. In a mouse study that we conducted on the toxicity of acetaminophen, a classical hepatotoxic substance, perturbations were found in seven other organs as well.^13^ This shows the power of organ-specific markers that enable us to see how organs actually communicate with each other as a network.

New emerging technologies are needed to explore and exploit the new dimensions of patient data space that is being created. In this article, three relevant issues will be discussed: 1) the integration of genetics and genomics through family genome sequencing; 2) the creation of mass spectrometry-based new assays that will enable the investigation of all human proteins; and 3) novel clinical assays that open new dimensions of patient data space.

## FAMILY GENOME SEQUENCING

The sequencing of families will be a revolutionary tool for medicine and human genetics in the future. The first family that we sequenced was a family of four in which the parents were normal but each of the children had a different genetic disease.^16^ Our initial hypothesis was that by sequencing the genome of all four members of this family we would be able to reduce the number of candidate genes for the genetic diseases in a significant manner. However, in reality we were able to do much more. By sequencing this family and other families, we were able to use family genome sequencing to eliminate more than 70% of the sequencing errors in a family of four, and 90% of the errors in a family of six. In addition, we were able to immediately identify rare variants because they were present in two or more members of the family and hence were very unlikely to be sequencing errors. This is important since it is the rare variants that are the origins of many diseases. Moreover, we could actually delineate the haplotypes of all the members of the family with enormous precision.^17^

**Figure 9.**
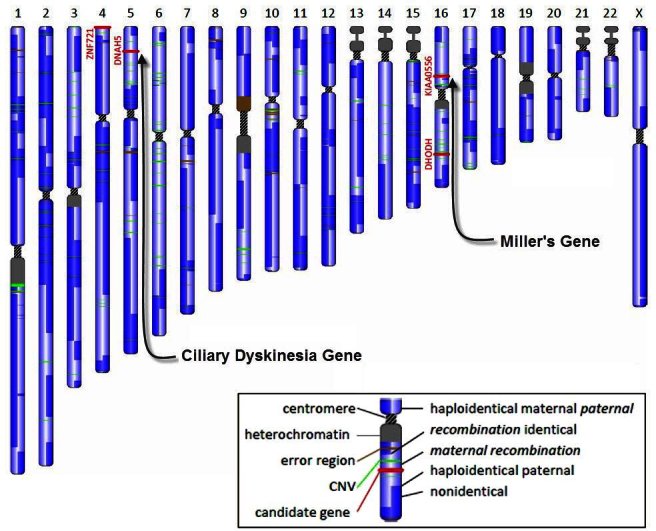
**A chromosomal map of one of the offspring with a genetic disease, showing possible candidate genes for the disease.**

The importance of the family genomics tool is in its ability to reduce significantly the dimensionality of chromosomal search space for disease genes. When searching for the disease genes, we can simply detect the haplotype blocks that the diseased individuals share which differ from the normal individuals and know that the disease genes must reside in these regions. In one such family we reduced the search space to 0.1% (J. Roach, personal communication). This vast reduction allows researchers to sort through the genes in the remaining DNA. In the previously mentioned family of four, we were able to identify four diseased gene candidates, and it was relatively easy to identify the disease genes encoding each of the two diseases (Figure 9).

In the near future, family sequencing will provide a fundamental medical record for each of us. The cost of sequencing is steadily decreasing, and within 5 years it will be well under $1,000. Third-generation sequencing technologies, using single-molecule physical measurements, will allow us to read sequences in lengths of 10,000 to 100,000 base-pairs at a time.^18^ Consequently, the speed of sequencing a human genome will be very rapid (e.g. 15 minutes), and the cost will be under $500.

All individuals will benefit from sequencing their genome. The benefit is in the identification of *actionable gene variants*. Actionable gene variants are defective genes which cause negative health effects, and medical intervention is available for reversing these effects. For example, if sequencing reveals a defect in a vitamin D transporter which has caused early onset of osteoporosis, an available solution would be taking megadoses of vitamin D to reverse the osteoporosis. We have identified almost 300 highly penetrant variants that fall into the actionable gene variants category. Sequencing the genome is a one-time investment, and once a genome is sequenced it can be searched every year for newly identified actionable genes. Sequencing is a smart investment in improving and optimizing wellness and avoiding disease.

### Proteomics

Over the past two years we have developed assays for about 20,000 human proteins which will be used for the organ-specific protein marker database, using a technique called *targeted mass spectrometry proteomics*.^14^,^19^,^20^ This technique allows us to assay 1–200 proteins an hour at the mid-attomole level, using minuscule amounts of blood. In 10 years’ time, we will be able to analyze massive numbers of patients, using ELISA assays with microfluidic technologies which are currently being developed.^21^–^24^ In addition, we have collaborated with researchers from Caltech for the last four years in creating a series of technologies whereby ELISA assays can be done on protein chips. We now have chips that can perform 20 ELISA assays on a fraction of a drop of blood in only a few minutes.^25^–^28^ Such chips are already being used in hospitals to assess cancer treatments as to how the patients respond to various drugs.^27^,^28^ Our ultimate goal is to use this chip technology to identify 50 organ-specific blood proteins from each of the 50 major organs and be able to quantify them from a drop of blood in a very short period of time. This will allow us to follow any transitions from health into disease for most of our major organ systems for each individual patient.

### Single Cell Analysis

J. Heath at Caltech is currently developing a microfluidic device that will be able to take a blood sample, isolate the white blood cells, and divide those cells into their 10 discrete populations. We can then investigate each separate cell type regarding its transcriptomes and proteomes.^27^,^28^ White cells that are separated in this manner can be as powerful a diagnostic for general phenomena, inflammation, immune responses, and other biological responses as the organ-specific blood proteins mentioned earlier.

**Figure 10.**
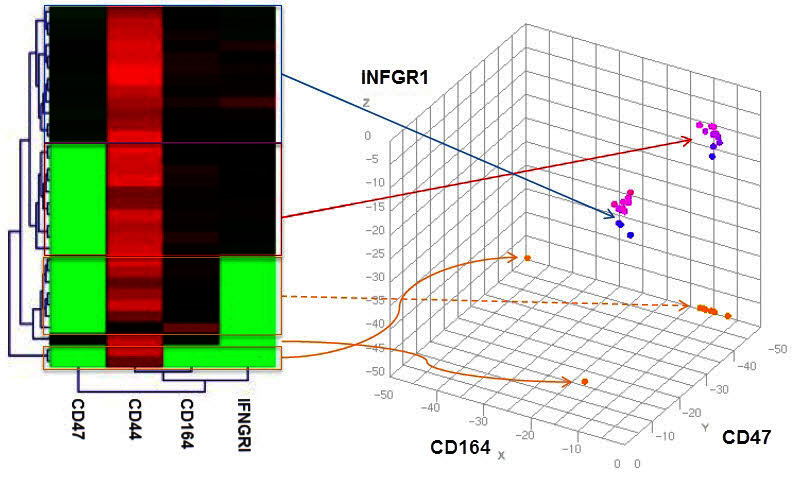
**A single cell analysis of 32 glioblastoma cells shows that their transcriptomes cluster to three distinct quantized groups.** Quantitative transcriptome clustering of single cells from the human glioblastoma cell line U87 (from L. Chen and Q Tian, personal communication).

Single cell analysis performed at our institute has shown that cancer cell lines have quantized cell populations (L. Chen and Q. Chen, personal communication). We took individual cells from a human glioblastoma cell line and performed single cell analysis. We examined 32 cells, quantified 24 different transcripts, and then mapped them in multi-dimensional space according to the quantification of their transcriptomes (Figure 10). Three discretely focused quantized populations were identified which included 30 of the 32 examined cells. We have no idea what the biological significance of these three quantized clusters is, but, if a whole tumor is homogenized and sequenced, the signal is lost in the noise. More recently we have examined single cells from a single glioblastoma tumor and confirmed the existence *in vivo* of quantized cell populations. Looking at single cell analyses for cancer and other diseases will be essential in the future.^29^,^30^ This type of single cell analysis can also be done at the protein level as well as at the transcript level. In collaboration with Heath, we developed single cell proteomics. Heath has been able to look at 10,000 individual cells and quantify approximately 20 secreted proteins per cell in a relatively short period of time.^27^,^28^

### Induced Pluripotent Stem Cells

Induced pluripotent stem (iPS) cells can be taken from different sources, such as fibroblasts and white blood cells, and can be amplified indefinitely. Cellular Dynamics, a stem cell company, can routinely create iPS cells from white blood cells and then differentiate them into four types of cells: neurons, cardiomyocytes, endothelial cells, and hepatocytes (see Cellular Dynamics, Inc., www.cellulardynamics.com) that are 99% pure.

We are planning on using single cell analysis to study the entire neuronal differentiation process. We will analyze them at eight different time points during differentiation, identify the quantized cell populations by single cell analyses, and then do a complete omics analysis on each of the quantized populations. In order to do such studies, we need very large numbers of starting cells, and that we can get from the large populations of iPS cells that can be differentiated into one of these four cellular phenotypes.

We are also planning to create iPS cells from patients with neurodegenerative disease and then differentiate patients’ iPS cells into neurons *in vitro*. We will then attempt stratification of complex diseases like Alzheimer’s into their discrete subtypes. We have recruited families for studying this disease. The differentiation process will provide most of the major classes of neurons, and the cells will be sorted by advanced cell sorting techniques. We plan to investigate each of those quantized neuron populations through various environmental signals, ligands, RNAi, and drugs. The hypothesis is that each quantized aspect of Alzheimer’s disease will have a different combination of disease-perturbed networks. Hence, the signals of each group will be different from each other and will uniquely identify the specific type of Alzheimer’s. Once that is accomplished, family genome sequencing will be performed to genetically stratify Alzheimer’s into different types of diseases. Subsequently, we will approach drug companies with the stratification data and request that they test the different drugs currently available for Alzheimer’s on specific subtypes of the disease. Our hope is that specific drugs will be more efficacious on one or more specific subtypes of the disease, thus providing better outcomes for the patients.

## P4 MEDICINE

As mentioned earlier, P4 medicine consists of predictive, preventive, personalized, and participatory medicine.^3^,^7^,^31^ P4 is a result of two convergences: systems medicine and the digital revolution. This article has so far focused on systems medicine and biology. The digital revolution has contributed to P4 medicine in three ways: the ability to deal with big data sets, the creation of social and business networks, and the creation of digital personal devices that will allow us to quantify parameters of health for ourselves. I will briefly discuss my predictions for the four Ps of P4 medicine in the future.^7^,^31^

### Predictive

Within the next 10 years, we should be able to sequence entire genomes in less than an hour’s time at the cost of a few hundred dollars. This will provide crucial insights into optimizing our wellness. In 10 years, we may have a little hand-held device that will prick your finger, make 2,500 blood measurements, and will longitudinally follow the organ-specific proteins for 50 different organs. This will allow us to detect many diseases at the earliest detectable phase, weeks, months, and maybe years before symptoms appear. In order to continue making advancements in systems medicine, I believe that all patient-derived data should be available to appropriate investigators for research purposes to continuously improve predictive medicine. After appropriate anonymization and strong protections against exploitation, society should have full access to patients’ data.

### Preventive

We will use drugs to push disease-perturbed networks back to their normal configurations, thus curing or ameloriating the disease. We are currently studying micro-organisms to determine the principles of re-engineering disease-perturbed networks with drugs and later will apply these principles to higher organisms. We should be able to use a systems approach for the immune system and finally get effective cellular immunity to create vaccines for AIDS and other diseases. So far, billions of dollars have been poured into vaccine research, but many of the immunization procedures that are used today are no different from what Jenner did in 1796 when he was credited with inventing vaccination.

One more important point about preventive medicine is that, instead of medicine focusing on disease as it does today, the focus in the future will be on wellness. Regular check-ups will allow the physician to longitudinally follow each patient and detect any perturbation that might lead to disease long before the onset of disease symptoms. In this manner, an individual’s wellness can be preserved without the disease state ever occurring.

### Personalized

We are all different. Our genomes are different, and our micro- and macroenvironments are different. In the future, diseases will be stratified according to the genetic make-up of the individual, and, in turn, treatments will be individually optimized. Individuals will be their own control in establishing a wellness baseline, monitoring the progression to disease state, and monitoring treatments that will perturb the systems back to a healthy state.

### Participatory

Patient-driven networks are going to be the driving force of this revolution in medicine. The health care community and especially physicians are by nature conservative, and therefore the push for change will be from the bottom up. Many of the large IT companies have difficulties comprehending the appropriate dimensionality of what will be needed to store and process the huge amount of new data that will be generated in the coming five years. Finally, how will we educate patients, physicians, and the health care community as to the benefits of P4 (systems) medicine? These education requirements pose a fascinating opportunity and different aspect of the IT for healthcare challenge.

## THE UNIQUENESS OF P4 MEDICINE

P4 medicine is in many ways different from the current practice of medicine. P4 medicine is proactive and uses an enormous number of measurements for diagnosis and treatment, for example genomic and proteomic data. P4 medicine focuses on the individual, especially regarding diagnostic tools and treatment options. The stratification of diseases will be key to approaching the FDA for approval on a specific drug with data on only 50 patients but with excellent response rates (say 95% or better).^7^,^31^ P4 medicine will probably be embraced by the public before it is embraced by the medical establishment. Therefore, the driving force will be the social networks. P4 medicine differs strikingly from the current “evidence-based” medicine in several regards (Table 1).

**Table 1.** **A comparison between evidence-based medicine and P4 medicine.**

**Reactive Medicine—Evidence-Based Medicine**	**Proactive P4 Medicine**
**Reactive**—respond after a patient is sick (symptoms-based)	**Proactive**—responds before a patient is sick (based on pre-symptomatic markers)
**Disease treatment** system	**Wellness maintenance** system
**Few measurements**	**Many measurements**, including complete genome sequencing, high-parameter blood diagnostics, many longitudinal omics measurements
**Disease-centric**, with standard of care associated with population-based disease diagnosis	**Individual-centric**, with standard of care tailored more fully to multiple measurements on the individual
**Records not highly linked nor data integrated**	**Deeply integrated data that can be mined for continued improvement of healthcare strategies**
**Large-scale diffusion** of medical information mediated mostly through physicians alone	**Social networking of patients** to enhanced shared experiences and diffusion of knowledge in consultation with their physicians
**Drugs tested against large populations**—tens of thousands to develop statistics for FDA	**Stratification of disease populations** into small groups, 50 or so, that can be effectively treated to achieve FDA approval

## IMPLEMENTING P4 MEDICINE

The essence of P4 medicine is the quantification of wellness and the demystification of disease. There are two challenges in bringing P4 medicine to the mainstream. The first challenge is the limitations of technologies. Technical advances are needed to provide the tools necessary for implementing P4 medicine. These tools are being invented and improved at ISB and at many other research institutions. The second challenge is that embodied in the fourth P—participatory. Societal changes must be implemented to facilitate a paradigm shift from the conventional evidence-based medicinal approach to personalized medicine’s predictive and preventive approach. These societal challenges include the following considerations: ethics, legal, privacy, patient data accessibility, who owns the data, etc.

To address the societal challenge, ISB has decided to create a limited number of strategic partnerships to bring P4 medicine to patients. One partnership is with the Grand Duchy of Luxembourg, where we are building an institute for systems medicine and helping the country with other programs. In return, we received $100 million over a five-year period to develop the strategies and tools of P4 medicine. We have also created the P4 Medicine Institute, a non-profit organization which in association with ISB is creating a network of clinical centers. We have two clinical centers, Ohio State and Peace Health, a community hospital system that has, together with ISB, agreed to explore creating a series of pilot projects that will demonstrate the preventative power of P4 medicine. Our target is to collaborate with six or so clinical centers. After demonstrating the P4 concept in this network of clinics, our next step will be to take P4 medicine to a small country and demonstrate its efficacy there.

## P4 MEDICINE, THE BIG PICTURE

P4 medicine will revolutionize the current evidence-based medicine in a number of ways. It will provide fundamental insights into disease mechanisms to enable diagnosis, therapy, and prevention for the individual patient. Blood will be the main window into the body to help diagnose disease, assess efficacy and toxicity of drugs, and assess wellness. The notion of stratifying diseases to distinct subtypes will allow the physician to target the therapy to the specific disease type, thus achieving far better outcomes. Patients will also be stratified into subgroups according to their responses to environmental challenges such as drugs, toxins, infectious disease agents, and poisons. P4 medicine will enable a multi-organ integrated approach to investigating diseases and, in addition, will facilitate a new approach to drug target discovery. By locating the networks that are perturbed by the disease state, drugs will be designed to perturb these networks in the opposite direction, thus promoting health. Lastly and most importantly, tools will be created for quantifying parameters and optimizing wellness.^7^,^31^

P4 medicine will cause every single sector of the health care community to rewrite their business plans, and many will be unable to do so due to their conservative business outlook. P4 medicine will create enormous wealth for those who adopt it. In 10–15 years, the wellness industry will far exceed the disease industry, also known as the health care industry. In addition, the wellness industry will probably be developed by companies that are completely different from those currently engaged in health care. P4 medicine will be able to reduce sharply the escalating costs of health care to the point where we will be able to export it to the developing world, leading to a democratization of health care, a concept unimaginable five years ago.

## CONCLUSION

Biology is a complex system. P4 medicine, along with systems biology, has forced researchers to collaborate in new unprecedented ways to develop the appropriate tools to deal with the complexities of biology and disease. The key is to attack the “big science problem” of health care with a systems-driven, integrative, cross-disciplinary, and milestone-driven ISB-like platform and culture. Small science, individual investigators, and their laboratories will play an important role in deciphering the complex details of the broad pictures that are painted by systems biology and systems medicine. The ultimate objectives of P4 medicine are simple: 1) improve health care, 2) reduce the cost of health care, and 3) stimulate innovation and new company creation.

However, biology and medicine are not the only complex systems problems that society is struggling with. All the major problems in society, for example health care, energy, environment, nutrition, and agriculture, are susceptible to the same kind of integrative systems approach which has been presented here. Therefore, institutions that create an appropriate systems-driven, cross-disciplinary, integrative, and milestone-driven environment for researching complex or big science problems will be uniquely positioned to transform and revolutionize the deciphering of many of society’s current challenges. The question of how academic, industrial, and governmental institutions will accept, build, and deploy these systems-driven and cross-disciplinary infrastructures is a fascinating one.

## Abbreviations:

iPS: induced pluripotent stem;
ISB: Institute for Systems Biology;
MBT: molecular biotechnology;
P4: predictive, preventive, personalized, and participatory.
